# Association between estimated pulse wave velocity and 28-day all-cause mortality in septic patients: a multicenter retrospective study

**DOI:** 10.1186/s12879-026-14047-2

**Published:** 2026-07-20

**Authors:** Lijuan Zhang, Wenwu Sun, Shu Wang, Bing Zhao, Yanxia Zhong, Yanyan Wang, Li Yang, Yujun Wang

**Affiliations:** 1 Department of Intensive Care Unit, The Central Hospital of Wuhan, Tongji Medical College, Huazhong University of Science and Technology, 26 Shengli Street, Wuhan, 430014 China; 2 Department of Emergency Medicine, Daping Hospital, Army Medical University, State Key Laboratory of Trauma and Chemical Poisoning, Chongqing, 400042 China; 3 Department of Intensive Care Medicine, Chongqing University Central Hospital, Chongqing Emergency Medical Center, Chongqing, 400016 China; 4 Department of Emergency, Ruijin Hospital, Shanghai Jiao Tong University School of Medicine, Shanghai, 200025 P. R. China

**Keywords:** Estimated pulse wave velocity, Sepsis, Mortality, Multicenter retrospective study

## Abstract

**Background:**

Estimated pulse wave velocity (ePWV) is an independent predictor of cardiovascular mortality and a marker of vascular stiffness; however, its association with mortality among septic patients remains unclear. Thus, this study aims to clarify the relationship between ePWV at admission and 28-day all-cause mortality (ACM) in septic patients.

**Methods:**

This retrospective study analyzed septic patients from three Chinese academic centers between September 2015 and July 2023. Patients were categorized into quartiles based on their admission ePWV. The primary outcome was 28-day ACM. Survival differences were assessed using Kaplan-Meier curves. Cox proportional hazards regression and restricted cubic splines (RCS) were employed to analyze the association between ePWV and 28-day ACM. Subgroup and interaction analyses were used to evaluate the robustness of the findings.

**Results:**

A total of 7702 patients were included (median age: 70 years), with 4635 (60.2%) males. Kaplan-Meier analysis showed that patients with higher ePWV had significantly lower survival (log-rank *P* < 0.001). After full adjustment, elevated ePWV was independently associated with 28-day ACM (hazard ratio [HR] per unit: 1.105, 95% confidence interval [CI]: 1.038–1.177), with the highest quartile exhibiting a more than two-fold increased risk compared with the lowest (HR: 2.320, 95% CI: 1.438–3.743; *P* < 0.001 for both). RCS analysis revealed a generally positive trend, with mortality risk increasing progressively across most of the ePWV range. According to interaction tests, the association was consistent across most subgroups, with significant interactions for mechanical ventilation and heart failure.

**Conclusions:**

Elevated ePWV at admission is a robust, independent predictor of 28-day ACM in septic patients. It likely reflects a combination of chronic vascular aging and acute sepsis–induced vascular dysfunction, serving as a valuable biomarker for early risk stratification and a potential indicator of a vascular-dominant sepsis phenotype.

**Supplementary Information:**

The online version contains supplementary material available at 10.1186/s12879-026-14047-2.

## Introduction

Sepsis, which is characterized by life-threatening organ dysfunction due to an abnormal host response to infection, presents a major global health challenge [1]. Annually, there are an estimated 48.9 million sepsis cases and 11 million related deaths worldwide [2]. A Chinese study found that septic patients constituted 20.6% of intensive care unit (ICU) admissions, demonstrating a 35.5% 90-day mortality [3]. Additionally, some survivors of sepsis might suffer from post-sepsis syndrome, defined as complications related to physical, cognitive, and psychological consequences of sepsis [4]. Thus, a reliable and easily obtainable clinical index to evaluate prognosis in patients with sepsis is urgently needed.

Estimated pulse wave velocity (ePWV), which has emerged as a novel indicator of large artery stiffening, can be calculated directly from age and average arterial blood pressure. Despite being a pressure-dependent mathematical approximation rather than a direct measure of intrinsic arterial stiffness, such as the gold-standard carotid-femoral PWV (cfPWV), ePWV shows reasonable correlation with cfPWV in stable populations, establishing its utility as a surrogate marker in clinical research [5]. Consequently, it serves as a crucial predictor for cardiovascular diseases and all-cause mortality (ACM) [5–9]. Recent studies have linked ePWV to several diseases, demonstrating increased ACM in acute kidney injury and coronary artery disease, as well as heightened risks of diabetes and stroke [10–13].

In sepsis, endothelial injury and loss of vascular autoregulation contribute to micro thrombosis, hypoperfusion, and multi-organ failure [14]. Some findings indicate that acute systemic inflammation increases arterial stiffness, potentially hindering hemodynamic adaptation and deteriorating organ perfusion [15]. Notably, patients with severe sepsis and septic shock in intensive care exhibit greater arterial stiffness compared with their healthy counterparts [16]. Although the pathophysiology of vascular dysfunction in sepsis is increasingly recognized, the prognostic value of non-invasive arterial stiffness assessment—specifically ePWV—in septic patients remains uncertain. Given this knowledge gap, this study aims to clarify the association between ePWV and mortality in sepsis, utilizing a multicenter cohort from three Chinese academic centers.

## Methods

### Participants

This retrospective study was conducted at three institutions: the Central Hospital of Wuhan (affiliated with Tongji Medical College, Huazhong University of Science and Technology), the Chongqing Emergency Medical Centre (affiliated with Chongqing University Central Hospital) and Ruijin Hospital (affiliated with Shanghai Jiaotong University School of Medicine). Ethical approval for this study was granted by the Ethics Committee of the Central Hospital of Wuhan (approval No. 2023-097-02), the Chongqing Emergency Medical Centre (approval No. 2025-24) and the Ruijin Hospital (approval No. 2025-138). Additionally, the requirement for informed consent was waived by the Ethics Committees of each participating institution due to the study’s non-interventional and retrospective nature. Data analysis adhered to the principles outlined in the 1964 Declaration of Helsinki.

The study screened consecutive adult patients diagnosed with sepsis from July 2017 to June 2023 at the Central Hospital of Wuhan, September 2015 to May 2022 at the Ruijin Hospital and January 2020 to July 2023 at the Chongqing Emergency Medical Centre. All patients received treatment according to established guidelines, including the prompt administration of antibiotics, initiation of fluid resuscitation and implementation of source control measures. Exclusion criteria for patient selection were (1) lack of MAP information, (2) death after withdrawal of treatment and (3) discharge with an unknown outcome.

### Definition, MAP and ePWV measurement

Sepsis was diagnosed based on the Sepsis 3.0 definition. The mean arterial pressure (MAP) was calculated using the following formula employing non-invasive blood pressure measurements: MAP = DBP + 0.4 × (SBP – DBP) (SBP, systolic blood pressure; DBP, diastolic blood pressure) [17]. The ePWV was determined using the formula: ePWV = 9.587–0.402 × age + 4.560 × 10^(− 3) × age^2–2.621 × 10^(− 5) × age^2 × MAP + 3.176 × 10^(− 3) × age × MAP – 1.832 × 10^(− 2) × MAP [18].

### Data collection and outcomes

Clinical variables were obtained from paper-based and digital medical records for each patient. Baseline demographic data included age, gender, body mass index (BMI) and comorbidities such as history of hypertension, diabetes, heart failure (HF), acute coronary syndrome, cirrhosis, chronic kidney disease, cerebrovascular disease, chronic pulmonary disease, malignancy, or immunosuppression. Vital signs, comprising SBP, DBP, respiratory rate, temperature, oxygen saturation (SpO2) and heart rate, were recorded as the first measurement within 6 h of ICU admission before any significant interventions. The median time from ICU admission to BP measurement was 0.5 h. In our cohort, 70.5% of the measurements were obtained before the initiation of vasopressors. Laboratory indicators collected within 48 h of admission included counts of white blood cells (WBC), lymphocytes (LYM) and platelets; levels of hemoglobin, C-reactive protein, blood urea nitrogen (BUN), creatinine, total bilirubin, alanine aminotransferase (ALT), aspartate aminotransferase (AST) and albumin; and measurements of prothrombin time (PT), activated partial thromboplastin time (APTT), D-dimer and lactate. The sequential organ failure assessment (SOFA) scores were evaluated within the first 24 h of admission. Additionally, mechanical ventilation and vasopressor use on the first day were documented. The primary outcome measure was 28-day ACM, with 90-day mortality, in-hospital mortality and hospitalization length as secondary outcomes.

### Statistical analysis

Baseline characteristic differences among groups were assessed using the Kruskal-Wallis test for skewed distributions and one-way ANOVA for normally distributed continuous variables. Categorical variables were analyzed using either the Chi-squared test or Fisher’s exact test. The Kaplan-Meier survival analysis examined endpoint event incidence across varying ePWV levels, with intergroup differences evaluated by the log-rank test. A univariate Cox regression analysis was conducted to determine risk factors linked to 28-day ACM. Three Cox proportional hazards models were used to estimate the hazard ratio (HR) and 95% confidence interval (CI) between ePWV and the endpoint. Covariate adjustments were informed by univariate Cox regression analysis (*P* < 0.1 for entry) and clinical relevance. Model 1 accounted for gender and age, whereas Model 2 was adjusted for age, gender, SOFA scores, specific comorbidities and laboratory test results. We adjusted for comorbidities such as HF, coronary heart disease and cerebrovascular disease due to their strong link to mortality and their role as indicators of vascular stiffness and reduced physiological reserve in sepsis. This adjustment helps control the confounding impact of cardiovascular burden on the ePWV-mortality relationship. Similarly, SOFA scores and lab markers were included for their known prognosti value in sepsis and potential confounding effects. The study utilized restricted cubic splines (RCS) to analyse ePWV as a continuous variable, clarifying the dose-response relationships with the primary outcome risk. Further analysis was performed for nonlinear associations. Subsequent stratified analyses assessed variations in ePWV’s effect on 28-day ACM across various subgroups. Results were presented as HRs with 95% CIs. Statistical analyses were performed using R software (version 4.5.0), with a two-sided significance threshold set at *P* = 0.05.

## Results

### Baseline characteristics of the study patients

**Fig. 1:**
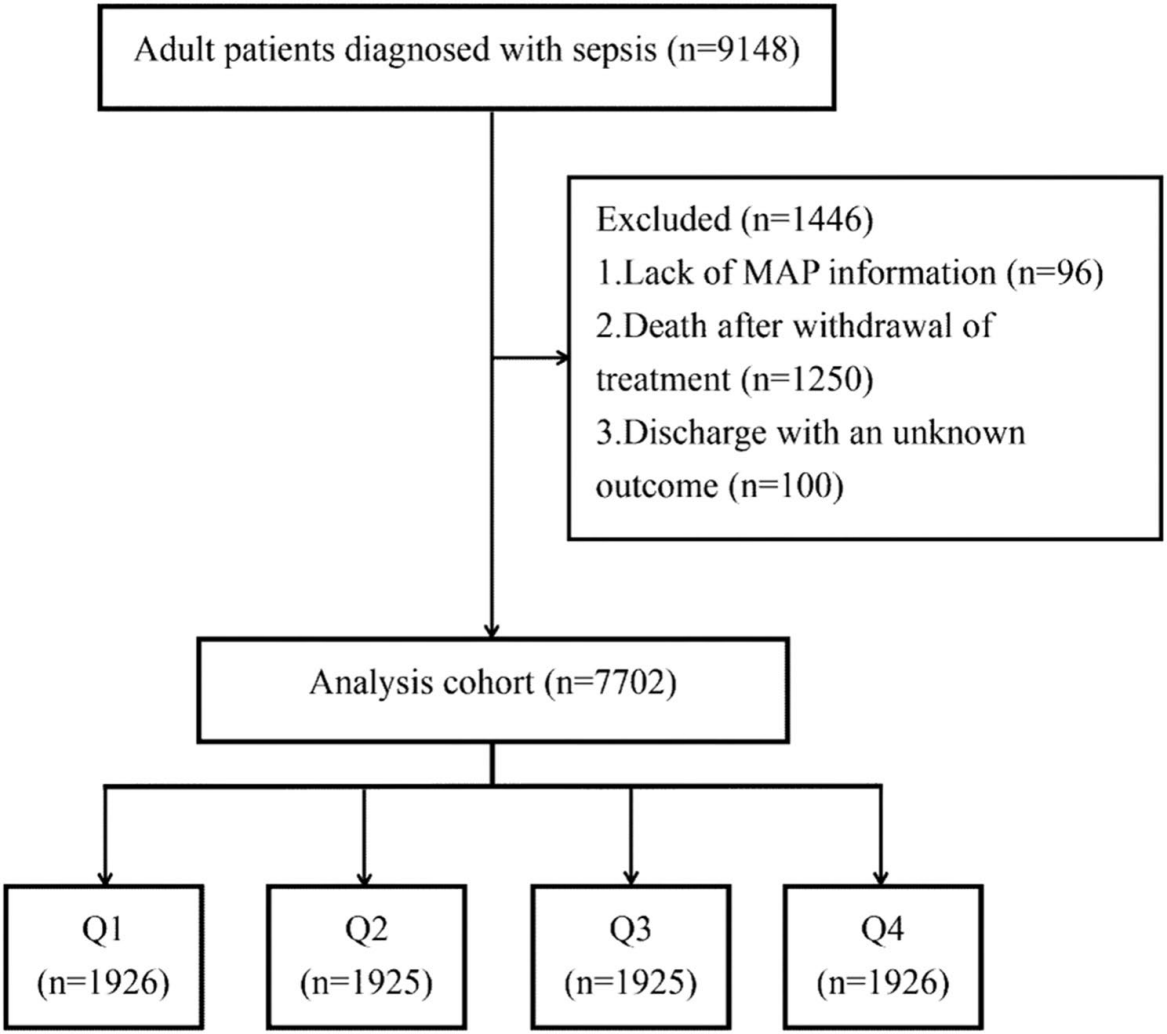
Selection of the study population

The study included a cohort of 7,702 patients diagnosed with sepsis, with the screening method illustrated in Fig. 1. The patients had a median age of 70 years (interquartile range [IQR]: 59–81), and 60.2% of them were males. This cohort was characterized by 14.4% in-hospital mortality, 11.2% 28-day mortality and 13.8% 90-day mortality. The median hospitalization length was 14 days. The patients were divided into four quartiles by ePWV levels: quartile 1 (Q1, ePWV ≤ 8.8653, *n* = 1,926), quartile 2 (Q2, 8.8653 < ePWV ≤ 11.0055, *n* = 1,925), quartile 3 (Q3, 11.0055 < ePWV ≤ 13.4230, *n* = 1,925) and quartile 4 (Q4, ePWV > 13.4230, *n* = 1,926). Subsequently, the distribution of variables across these quartiles was analyzed. Table 1 provides a comprehensive overview of all baseline characteristics.

**Table 1:** Characteristics and outcomes of participants grouped according to ePWV

		No (%)/Median (IQR)				*P*-value
	Total (*n* = 7702)	Q1 (*n* = 1926)	Q2 (*n* = 1925)	Q3 (*n* = 1925)	Q4 (*n* = 1926)	
**Age**	70 (59–81)	51 (39–58)	65 (60–70)	75 (71–80)	87 (83–90)	**< 0.001**
**Male**	4635 (60.2%)	1131 (58.7%)	1193 (62.0%)	1167 (60.6%)	1144 (59.4%)	0.176
**BMI**	22.5 (19.6, 24.8)	22.5 (20.1,25.4)	22.8 (20.0,25.1)	22.5 (20.0,24.8)	21.5 (18.8,24.2)	**< 0.001**
**Score system**						
SOFA	3 (2–5)	3 (2–6)	3 (2–5)	3 (2–5)	3 (2–5)	**< 0.001**
**Comorbidities**						
Hypertension	3706 (48.1%)	411 (21.3%)	849 (44.1%)	1138 (59.1%)	1308 (67.9%)	**< 0.001**
Diabetes	2550 (33.1%)	450 (23.4%)	717 (37.3%)	727 (37.8%)	656 (34.1%)	**< 0.001**
Heart failure	1092 (14.2%)	91 (4.7%)	194 (10.1%)	289 (15.0%)	518 (26.9%)	**< 0.001**
Acute coronary syndrome	679 (8.8%)	62 (3.2%)	137 (7.1%)	183 (9.5%)	297 (15.4%)	**< 0.001**
Cirrhosis	254 (3.3%)	90 (4.7%)	74 (3.8%)	51 (2.7%)	39 (2.0%)	**< 0.001**
Chronic kidney disease	793 (10.3%)	110 (5.7%)	169 (8.8%)	219 (11.4%)	295 (15.3%)	**< 0.001**
Cerebrovascular disease	1683 (21.9%)	182 (9.5%)	337 (17.5%)	519 (27.0%)	645 (33.5%)	**< 0.001**
Chronic pulmonary disease	1012 (13.1%)	96 (5.0%)	203 (10.5%)	308 (16.0%)	405 (21.0%)	**< 0.001**
Malignancy or immunosuppression	1235 (16.0%)	281 (14.6%)	340 (17.7%)	356 (18.5%)	258 (13.4%)	**< 0.001**
**Admission vital signs**						
SBP (mmHg)	125 (109–142)	110 (96–124)	124 (110–136)	131 (115–146)	138 (121–157)	**< 0.001**
DBP (mmHg)	72 (62–81)	66 (55–76)	73 (63–80)	74 (64–82)	76 (65–85)	**< 0.001**
Heart rate (beats/min)	93 (80–109)	98 (82–115)	92 (80–108)	92 (80–107)	90 (80–105)	**< 0.001**
Respiratory rate (breaths/min)	20 (18–23)	20 (18–22)	20 (18–22)	20 (18–23)	20 (20–23)	**< 0.001**
Temperature (℃)	36.8 (36.5–37.7)	36.8 (36.5–38.0)	36.8 (36.5–37.8)	36.8 (36.5–37.6)	36.7 (36.5–37.5)	**< 0.001**
**Admission therapies**						
Mechanical ventilation	1008 (13.1%)	230 (11.9%)	251 (13.0%)	276 (14.3%)	251 (13.0%)	0.053
Vasopressor	434 (22.6%)	100 (5.2%)	83 (4.3%)	67 (3.5%)	184 (9.6%)	**< 0.001**
**Admission laboratory results**						
WBC (10^9^ /L)	10.3 (6.9–15.2)	10.6 (6.8–16.0)	10.1 (6.6–15.2)	10.4 (6.9–15.2)	10.1 (7.1–14.4)	0.122
LYM (10^9^ /L)	0.78 (0.47–1.25)	0.85 (0.51–1.40)	0.82 (0.49–1.27)	0.74 (0.44–1.16)	0.74 (0.45–1.17)	**< 0.001**
Platelet (10^9^ /L)	176 (117–247)	175 (107–247)	173 (112–250)	179 (121–249)	177 (126–242)	0.118
Hemoglobin (g/L)	113 (95–130)	115 (94–133)	114 (95–131)	113 (95–129)	111 (94–128)	**0.006**
C-reactive protein (mg/dL)	49.9 (13.1, 124.0)	54.9 (13.8-139.6)	53.0 (14.0-131.1)	50.8 (14.2-123.5)	44.1 (11.6-108.2)	**< 0.001**
Procalcitonin (ng/mL)	1.47 (0.24–11.46)	2.62 (0.35–20.10)	1.66 (0.25–12.95)	1.69 (0.25–11.31)	0.85 (0.18–6.45)	**< 0.001**
Blood glucose (mmol/L)	7.4 (5.8–10.7)	6.9 (5.4–10.0)	7.4 (5.7–11.1)	7.7 (6.0-10.8)	7.7 (6.1–10.5)	**< 0.001**
BUN (mmol/L)	7.8 (5.3–12.5)	6.3 (4.3–10.9)	7.3 (5.0-11.8)	8.2 (5.8–12.9)	9.1 (6.3–14.5)	**< 0.001**
Creatinine (µmol/L)	87 (64–139)	82 (60–132)	83 (63–131)	89 (65–141)	94 (68–149)	**< 0.001**
Total bilirubin (mmol/L)	13.0 (8.6–20.6)	13.8 (9.2–22.8)	13.2 (8.8–21.2)	12.9 (8.4–20.6)	12.2 (8.2–18.2)	**< 0.001**
ALT (U/L)	22 (13–42)	26 (14–58)	23 (14–44)	20 (12–39)	18 (12–33)	**< 0.001**
AST (U/L)	28 (18–54)	31 (18–66)	28 (18–54)	28 (18–51)	27 (18–48)	**< 0.001**
Albumin (g/L)	32.4 (27.8–37.3)	32.6 (27.4–38.7)	32.0 (27.4–37.4)	32.0 (27.6–37.0)	32.7 (28.6–37.0)	**0.023**
PT (s)	13.8 (12.6–15.2)	13.8 (12.6–15.5)	13.6 (12.3–15.1)	13.7 (12.5–15.0)	13.9 (12.9–15.2)	**< 0.001**
APTT (s)	34.8 (29.9–41.0)	35.3 (30.2–41.7)	33.9 (29.0-40.4)	34.4 (29.6–40.4)	35.6 (30.9–41.1)	**< 0.001**
D-dimer (g/L)	2.43 (1.11–5.25)	2.50 (1.06–5.84)	2.29 (1.01–5.05)	2.48 (1.17–5.08)	2.44 (1.24–5.02)	**0.047**
lactate (mg/dL)	2.4 (1.7–3.6)	2.5 (1.6–4.1)	2.4 (1.6–3.6)	2.4 (1.6–3.4)	2.4 (1.7–3.5)	0.090
**Primary outcome**						
28-day mortality	862 (11.2%)	162 (8.4%)	189 (9.8%)	222 (11.5%)	289 (15.0%)	**< 0.001**
**Secondary outcome**						
90-day mortality	1059 (13.8%)	184 (9.6%)	228 (11.8%)	277 (14.4%)	370 (19.2%)	**< 0.001**
In-hospital mortality	1110 (14.4%)	190 (9.9%)	233 (12.1%)	284 (14.8%)	403 (20.9%)	**< 0.001**
Hospital length of stay (day)	14 (8–23)	12 (7–21)	14 (8–22)	14 (8–24)	16 (10–26)	**< 0.001**

Vasopressor use refers to any vasopressor administration within the first 24 h of ICU admission

Abbreviations: ePWV, estimated pulse wave velocity; BMI, body mass index; SOFA, sequential organ failure assessment; WBC, white blood cell count; LYM, lymphocyte count; BUN, blood urea nitrogen; ALT, alanine aminotransferase; AST, aspartate aminotransferase; PT, prothrombin time; APTT, activated partial thromboplastin time; IQR, interquartile range

Patients in the Q4 group were notably older than those in the Q1 group. All groups showed no significant differences in gender. Our study observed a progressive rise in certain comorbidities, including hypertension, HF, acute coronary syndrome, chronic kidney disease, cerebrovascular disease and chronic pulmonary disease from Q1 to Q4, whereas the number of comorbid cirrhosis cases showed a sequential decline. SBP, DBP, blood glucose, BUN, and creatinine exhibited an upward trend throughout Q1 to Q4, whereas heart rate, LYM, hemoglobin, C-reactive protein, total bilirubin, ALT, and AST demonstrated a downward trend. Furthermore, BMI, SOFA scores and comorbidities (diabetes, malignancy, or immunosuppression) demonstrated significant differences among the groups. Additionally, vital signs (respiratory rate and temperature), admission therapies (vasopressor use) and laboratory parameters (procalcitonin, albumin, PT, APTT, D-dimer and lactate) differed significantly (all *P* < 0.05). Patients in higher ePWV quartiles had significantly higher 28-day, 90-day, and in-hospital mortality, as well as longer hospitalization, compared with those in lower ePWV quartiles (*P* < 0.001).

### Primary outcomes

**Fig. 2:**
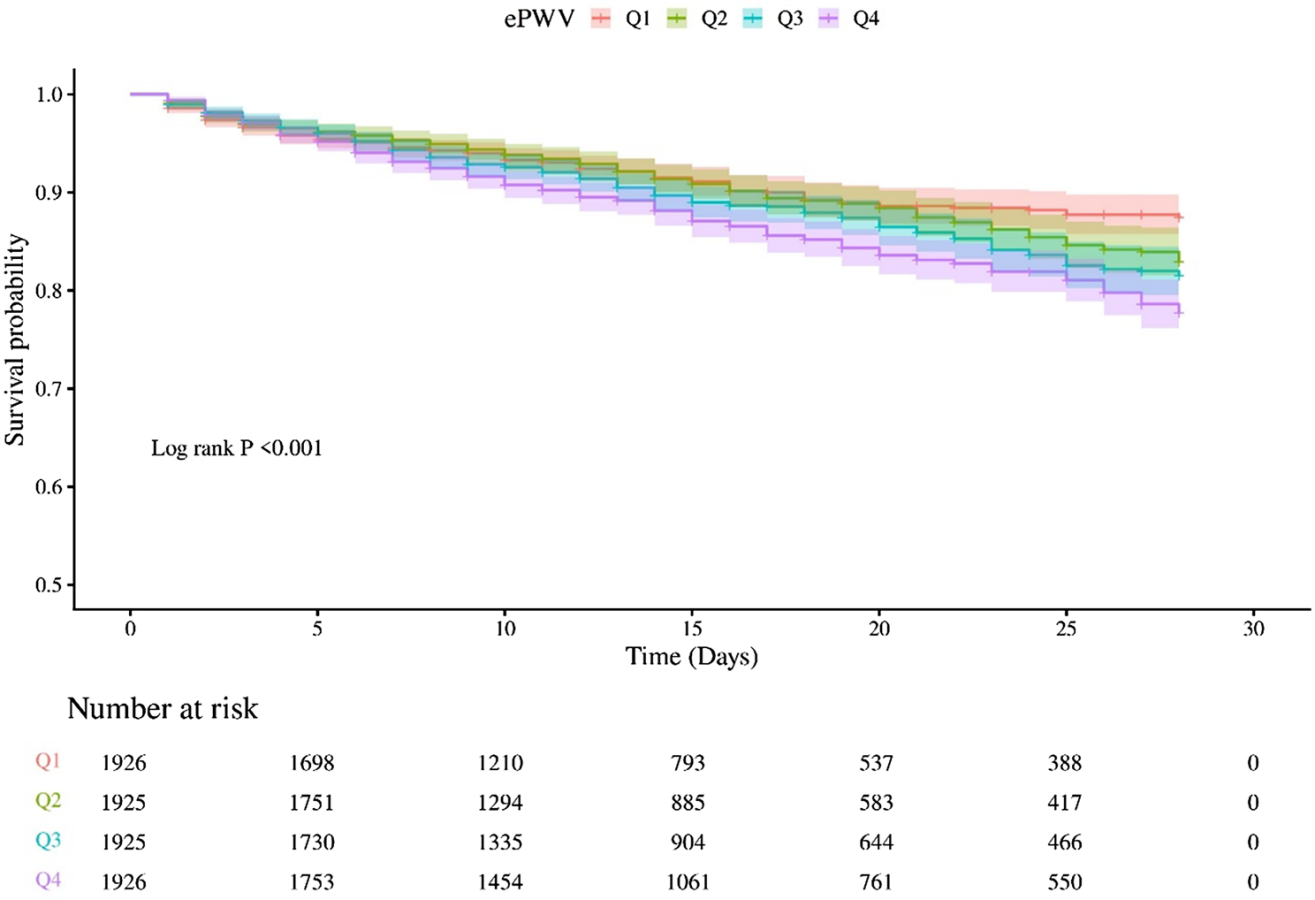
28-day Kaplan-Meier survival curve. Kaplan-Meier curves showing the survival rates at 28 days for each quartile. ePWV: quartile 1 (ePWV ≤ 8.8653), quartile 2 (8.8653 < ePWV ≤ 11.0055), quartile 3 (11.0055 < ePWV ≤ 13.4230) and quartile 4 (ePWV > 13.4230)

Figure 2 illustrates the Kaplan-Meier survival curves that show a continuous decline in 28-day survival probability for septic patients from Q1 to Q4. Significant intergroup differences in 28-day mortality were observed (log-rank test, *P* < 0.001).

### Relationship between ePWV and clinical outcomes of patients with sepsis

Table S1 presents the univariate Cox regression analysis results for the risk factors linked to 28-day ACM. In the non-adjusted model 1, higher ePWV levels were significantly linked to an increased risk of 28-day ACM (HR: 1.069, 95% CI: 1.043–1.095; *P* < 0.001). Three Cox proportional hazards regression models were used to determine the independent impact of ePWV on 28-day ACM, guided by univariate analysis and clinically relevant prognostic variables (Table 2). Notably, the association between ePWV and mortality shifted across models. In the initially adjusted Model 1 (adjusted for age and sex), the association inverted (HR: 0.901; 95% CI: 0.857–0.948; *P* < 0.001). However, following further adjustment for SOFA scores, comorbidities, and laboratory markers in the fully adjusted Model 2, the positive association re-emerged (HR: 1.105; 95% CI: 1.038–1.177; *P* < 0.001), indicating that ePWV is an independent predictor of mortality. Consistent with these findings, when treated as a categorical variable, patients in the highest ePWV quartile had a significantly lower 28-day ACM risk compared with the lowest quartile in Model 1 (HR: 0.651; 95% CI: 0.454–0.934; *P* < 0.001), whereas Model 2 showed a significantly higher risk (HR: 2.320; 95% CI: 1.438–3.743; *P* < 0.001).

**Table 2:** Cox proportional hazard ratios for all-cause mortality at 28 days

Categories	Non-adjusted model		Adjusted model 1		Adjusted model 2	
	HR (95% CI)	*P*-value	HR (95% CI)	*P*-value	HR (95% CI)	*P*-value
**Continuous ePWV per unit**	1.049 (1.027–1.072)	< 0.001	0.901 (0.857–0.948)	< 0.001	1.105 (1.038–1.177)	0.002
**Q1**	Reference	/	Reference	/	Reference	/
**Q2**	1.106 (0.896–1.364)	0.348	0.778 (0.612–0.990)	0.041	1.443 (1.034–2.015)	0.031
**Q3**	1.263 (1.032–1.547)	0.024	0.707 (0.531–0.942)	0.018	1.809 (1.230–2.662)	0.003
**Q4**	1.531 (1.262–1.856)	< 0.001	0.651 (0.454–0.934)	0.020	2.320 (1.438–3.743)	< 0.001
***P*** **for trend**	< 0.001		0.033		< 0.001	

Adjusted model 1, adjusted for age and gender. Adjusted model 2, adjusted for age, gender, comorbidities (heart failure, acute coronary syndrome, cerebrovascular disease, malignancy, or immunosuppression), SOFA and laboratory tests (WBC, platelet, hemoglobin, C-reactive protein, BUN, creatinine, ALT, AST, albumin, PT, APTT, D-dimer and lactate)

### The detection of nonlinear relationships

**Fig. 3:**
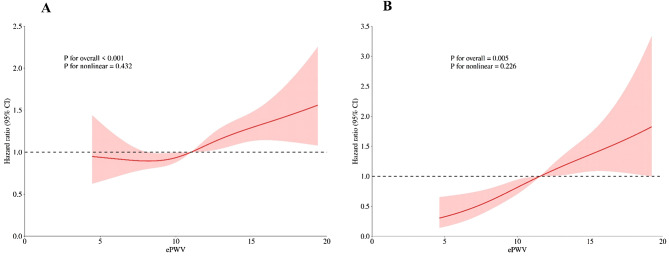
Restricted cubic spline curve for the ePWV hazard ratio for 28-day mortality. **A**: Unadjusted. **B**: adjusted for age, gender, comorbidities (heart failure, acute coronary syndrome, cerebrovascular disease, malignancy, or immunosuppression), SOFA and laboratory tests (WBC, platelet, hemoglobin, C-reactive protein, BUN, creatinine, ALT, AST, albumin, PT, APTT, D-dimer and lactate). Curves represent estimated adjusted hazard ratios, and shaded ribbons represent 95% confidence intervals. The horizontal dashed line represents a hazard ratio of 1.0. HR, hazard ratio; CI, confidence interval

We performed RCS analysis with four knots to further explore the potential nonlinear relationship (Fig. 3). A generally positive trend was observed between ePWV and 28-day mortality risk (Fig. 3A). The overall P-value was significant (P for overall < 0.001), whereas the P-value for nonlinearity was 0.432, indicating no significant deviation from linearity. After adjusting for confounders (Fig. 3B), this linear relationship remained robust (P for overall = 0.005; P for nonlinear = 0.226). Therefore, the risk of 28-day mortality increases steadily with rising ePWV, without an observable threshold effect.

### Subgroup analysis

**Fig. 4:**
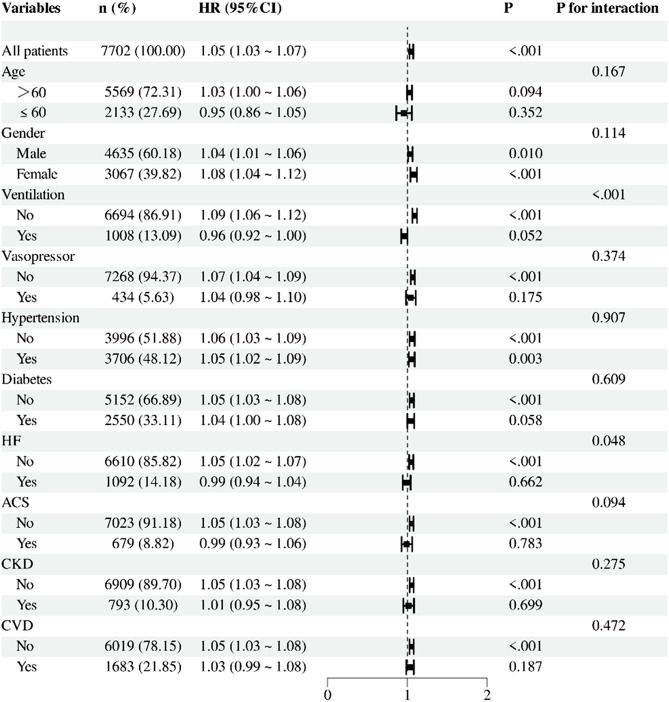
Forest plots of HR for the 28-day mortality in different subgroups. HR, hazard ratio; CI, confidence interval; HF, heart failure; ACS, acute coronary syndrome; CKD, chronic kidney disease; CVD, cerebrovascular disease

Subgroup analyses were performed to evaluate the consistency of the association between ePWV and 28-day mortality across different patient characteristics (Fig. 4). Overall, the positive association between higher ePWV and increased mortality risk was generally consistent across most subgroups. Mechanical ventilation (P for interaction < 0.001) and HF (P for interaction = 0.048) showed statistically significant interactions. Specifically, the association was significant in patients without mechanical ventilation (HR: 1.09, 95% CI: 1.06–1.12) and without HF (HR: 1.05, 95% CI: 1.02–1.07), but not in those requiring ventilation or with a history of HF. Notably, despite the confidence intervals crossing unity in certain subgroups (those aged ≤ 60 years, patients with acute coronary syndrome and chronic kidney diseases), the interaction tests were not significant.

## Discussion

To our knowledge, this study was a large-scale, multicenter retrospective investigation specifically evaluating the association between ePWV and 28-day ACM in a heterogeneous population of septic patients. Our primary finding is that elevated ePWV, serving as a surrogate marker for arterial stiffness, is independently and robustly associated with an increased risk of 28-day mortality in patients with sepsis. Notably, the direction of the association shifted after adjustment, indicating that age acted as a strong negative confounder; since older age correlates positively with both ePWV and mortality, adjusting for it was essential to unmask the true independent risk. To characterize the dose-response relationship, we employed restricted cubic splines (RCS), an approach consistent with recent methodological precedents in sepsis research [19]. The RCS analysis revealed a generally positive trend, with mortality risk increasing progressively across most of the ePWV range; however, wide confidence intervals at the lowest values preclude the identification of a clear safe threshold. Similar to composite markers such as the creatinine to albumin ratio or the glucose potassium ratio, ePWV may serve as an integrated vascular health index [20, 21]. Collectively, these findings highlight the potential of ePWV as a practical, non-invasive, and cost-effective tool for early risk stratification in the ICU, allowing medical personnel to identify a subset of septic patients with high vascular vulnerability who are at imminent risk of adverse outcomes.

Our results corroborate and extend a growing body of evidence that links arterial stiffness to adverse outcomes in critically ill patients. Although previous studies have firmly established ePWV as a predictor of cardiovascular events and mortality in the general population [8], patients with coronary artery disease [22, 23] and those with chronic kidney disease [24, 25], its role in acute infectious conditions has been unclear. Our findings align with recent publications on other acute inflammatory states, such as COVID-19, in which elevated arterial stiffness was linked to poor prognosis [26]. Crucially, our study bridges the gap between chronic vascular aging and acute sepsis pathophysiology. Although Kazune et al. demonstrated the prognostic value of directly measured cfPWV in septic shock, their study was limited by sample size and the technical complexity of tonometry [16]. Our study offers a more scalable solution for clinical practice by validating the utility of ePWV, which relies on routine hemodynamic parameters (age and blood pressure). Our study demonstrates that even without specialized equipment, the physiological signal of vascular stiffening remains a potent predictor of mortality, reinforcing the concept that vascular health is a critical determinant of survival in sepsis.

The observed association most probably reflects a complex interplay between chronic vascular remodeling and acute sepsis–induced dysfunction, a phenomenon we term the “double hit” (i.e., two sequential insults: chronic vascular aging then acute septic injury). During the “first hit,” a high baseline ePWV indicates pre-existing structural vascular aging. Patients with stiffer arteries have reduced physiological reserve, characterized by endothelial dysfunction and impaired vasodilatory capacity [27], compromising their ability to adapt to hemodynamic stress. Then, the acute phase of sepsis delivers the “second hit.” Consequently, a high ePWV at admission indicates compromised vascular health, signifying the “double hit” of reduced physiological reserve compounded by severe acute vascular injury [28, 29]. This mechanism effectively stratifies patients at risk of early mortality, explaining why elevated ePWV is independently associated with increased 28-day mortality.

Mechanistically, elevated arterial stiffness impairs the Windkessel effect of the aorta [30], increasing pulsatile stress and afterload on the left ventricle. This is particularly detrimental in sepsis, in which myocardial depression is common [31]. Furthermore, stiff arteries transmit excessive pulsatile energy into the microcirculation, potentially damaging delicate capillary beds in vital organs such as the kidneys and brain [32]. These “macro-micro” interactions suggest that ePWV serves as a functional indicator of the inability of the vascular system to buffer hemodynamic stress, leading to “occult hypoperfusion,” during which systemic blood pressure appears preserved, but tissue-level perfusion is critically compromised [33, 34].

Our subgroup analyses revealed significant interactions with mechanical ventilation and HF, providing novel insights into sepsis heterogeneity. Although the association between ePWV and mortality did not reach statistical significance within these subgroups (likely due to smaller sample sizes and wide confidence intervals), the significant interaction P-values (P for interaction < 0.05) indicate true effect modification. Consistent with standard epidemiological practice, we rely on the interaction tests to detect heterogeneity, while the within-stratum estimates should be interpreted with caution given the reduced precision in these subgroups. The predictive value of ePWV was attenuated in patients requiring mechanical ventilation or those with a history of HF. In the context of mechanical ventilation, positive pressure alters intrathoracic pressure and venous return, profoundly influencing ventricular-vascular coupling [35]. These iatrogenic hemodynamic changes may transiently mask the underlying arterial stiffness or introduce variability that weakens the prognostic signal of a single admission ePWV measurement [36]. Regarding HF, its pathophysiology is dominated by pump failure rather than pure vascular dysfunction. In patients with HF, the “ceiling effect” of neurohormonal activation and severe cardiac dysfunction may overshadow the additional risk contributed by arterial stiffness [37, 38]. In contrast, for subgroups such as age > 60 years and chronic kidney disease, although the confidence intervals crossed the null value, the formal tests for interaction were non‑significant (*P* > 0.05), indicating that the effect of ePWV in these populations is not fundamentally different from that in the overall cohort. Hence, ePWV is not a universal predictor for all shock subtypes but may be particularly valuable in identifying a “vasoplegic” or “vascular-dominant” phenotype of sepsis. In patients without severe baseline cardiac dysfunction, vascular stiffness represents the primary driver of mortality, potentially serving as a biomarker to guide vasopressor strategies or fluid management in future precision medicine trials.

The mathematical derivation of ePWV from MAP presents a key interpretive challenge. Although septic shock involves hypotension, higher ePWV predicted mortality. This is attributable to the exponential rise of ePWV with pressure in stiff vessels [39]. A ‘high pressure, high stiffness’ state, which is often sustained by high-dose vasopressors, reflects vascular rigidity and occult hypoperfusion. However, we acknowledge that hemodynamic interventions directly affect calculated ePWV. In our cohort, patients receiving vasopressors sometimes exhibited lower ePWV due to refractory hypotension, despite being a high-risk group. We posit that this time-varying complexity might have attenuated the observed association; therefore, the true predictive power of baseline arterial stiffness could be even greater.

### Limitations

This study has several limitations. First, we cannot establish a causal relationship between ePWV and 28-day ACM in septic patients due to the retrospective observational design, with our findings only providing correlational evidence. Second, although multicenter data were included, heterogeneity across sites and the limited sample size might have influenced the detection of subgroup differences (e.g., by infection source) as well as the assessment of the predictive value of ePWV for long-term prognosis. Third, the timing of ePWV measurements varied across centers. Furthermore, despite efforts to standardize data collection, measurement bias could still exist. Fourth, unaccounted time-varying confounders—such as vasoactive agent usage, dynamic hemodynamic changes, and the lack of systematically recorded serial blood pressure measurements—limit our ability to fully adjust for factors that might alter ePWV and its association with outcomes. This complexity poses a challenge for causal inference. Future studies should therefore employ the Target Trial Emulation framework to rigorously disentangle the causal effects of vasoactive therapies and dynamic arterial stiffness on outcomes, and should also examine whether serial ePWV measurements, rather than a single admission value, provide stronger prognostic information by capturing dynamic vascular changes during the course of sepsis.

## Conclusion

In conclusion, elevated ePWV at admission is a robust, independent predictor of 28-day mortality in septic patients. It likely reflects the “double hit” of chronic vascular aging and acute sepsis–induced vascular dysfunction. Although its prognostic performance varies in specific subgroups such as HF patients, ePWV remains a promising, accessible biomarker for identifying high-risk patients and characterizing the vascular phenotype of sepsis.

## Supplementary Information

Below is the link to the electronic supplementary material.

Supplementary Material 1: Table S1: Univariate Cox regression analysis of risk factors associated with 28-day mortality in patients with sepsis.

## Data Availability

Raw data supporting the obtained results are available at the corresponding author.

## Abbreviations

ePWV: Estimated pulse wave velocity
BMI: Body mass index
SOFA: Sequential organ failure assessment
WBC: White blood cell count
LYM: Lymphocyte count
BUN: Blood urea nitrogen
ALT: Alanine aminotransferase
AST: Aspartate aminotransferase
PT: Prothrombin time
APTT: Activated partial thromboplastin time
IQR: Interquartile range
