# Supplementary material for: Association between estimated pulse wave velocity and 28-day all-cause mortality in septic patients: a multicenter retrospective study
Source: BMC Infect Dis. 2026 Jul 20;26:1460. doi: 10.1186/s12879-026-14047-2 (PMC13459326; doi:10.1186/s12879-026-14047-2)
Supplement: Supplementary file 1 [file 12879_2026_14047_MOESM1_ESM.docx]

**SUPPLEMENTAL MATERIAL**

**Association Between Estimated Pulse Wave Velocity and 28-Day All-Cause Mortality in Septic Patients: A Multicenter Retrospective Study**

Lijuan Zhang; Wenwu Sun; Shu Wang; Bing Zhao; Yanxia Zhong; Yanyan Wang; Li Yang; Yujun Wang.

**Contents:**

**Table S1**: Univariate Cox regression analysis of risk factors associated with 28-day mortality in patients with sepsis

.

|  | **Univariable analysis** | |
| --- | --- | --- |
|  | **HR (95% CI)** | ***P-value*** |
| **ePWV** | 1.049 (1.027-1.072) | **<0.001** |
| **Age( ＞60 vs. 18-60)** | 1.526 (1.286-1.811) | **<0.001** |
| **Sex (male vs. female)** | 1.282 (1.112-1.479) | **<0.001** |
| **BMI** | 0.995 (0.979-1.011) | 0.503 |
| **Score system** |  |  |
| SOFA | 1.224 (1.202-1.246) | <0.001 |
| **Comorbidities** |  |  |
| Hypertension | 0.990 (0.866-1.132) | 0.885 |
| Diabetes | 0.938 (0.814-1.081) | 0.378 |
| Heart failure | 1.564 (1.328-1.841) | **<0.001** |
| Acute coronary syndrome | 1.402 (1.142-1.723) | **0.001** |
| Cirrhosis | 1.278 (0.915-1.784) | 0.150 |
| Chronic kidney disease | 1.132 (0.922-1.389) | 0.237 |
| Cerebrovascular disease | 1.149 (0.987-1.338) | **0.073** |
| Chronic pulmonary disease | 1.156 (0.958-1.396) | 0.131 |
| Malignancy or immunosuppression | 1.335 (1.133-1.574) | **<0.001** |
| **Admission laboratory results** |  |  |
| WBC (109 /L) | 1.011 (1.005-1.017) | **<0.001** |
| LYM (109 /L) | 0.941 (0.866-1.022) | 0.151 |
| Platelet (109 /L) | 0.998 (0.997-0.999) | **<0.001** |
| Hemoglobin (g/L) | 0.995 (0.992-0.997) | **<0.001** |
| C-reactive protein (mg/dL) | 1.002 (1.001-1.002) | **<0.001** |
| Procalcitonin (ng/mL) | 1.000 (1.000-1.000) | 0.421 |
| Blood glucose (mmol/L) | 1.009 (0.998-1.021) | 0.112 |
| BUN (mmol/L ) | 1.039 (1.034-1.044) | **<0.001** |
| Creatinine (μmol/L) | 1.001 (1.001-1.002) | **<0.001** |
| Total bilirubin (mmol/L) | 1.002 (1.001-1.003) | **<0.001** |
| ALT (U/L) | 1.001 (1.001-1.001) | **<0.001** |
| AST (U/L) | 1.001 (1.001-1.001) | **<0.001** |
| Albumin (g/L) | 0.950 (0.940-0.960) | **<0.001** |
| PT (s) | 1.032 (1.027-1.037) | **<0.001** |
| APTT (s) | 1.018 (1.015-1.021) | **<0.001** |
| D-dimer (g/L) | 1.015 (1.011-1.018) | **<0.001** |
| Lactate (mg/dL) | 1.124 (1.106-1.143) | **<0.001** |

Abbreviations: ePWV, estimated pulse wave velocity; BMI, body mass index; SOFA, sequential organ failure assessment; GCS, glasgow coma scale; WBC, white blood cell count; LYM, lymphocyte count; BUN, blood urea nitrogen; ALT, alanine aminotransferase; AST, aspartate aminotransferase; PT, prothrombin time; APTT, activated partial thromboplastin time; IQR, interquartile range.
